# Residual symptoms and the quality of life in individuals recovered from COVID-19 infection: A survey from Pakistan

**DOI:** 10.1016/j.amsu.2022.103361

**Published:** 2022-02-11

**Authors:** Mohammad Aadil Qamar, Russell Seth Martins, Rubaid Azhar Dhillon, Areeba Tharwani, Omar Irfan, Qosain Fatima Suriya, Wajiha Rizwan, Javaid Ahmed Khan, Ali bin Sarwar Zubairi

**Affiliations:** a Ziauddin University, Karachi, Pakistan; b Medical College, Aga Khan University, Karachi, Pakistan; c Medical College, Riphah International University, Rawalpindi, Pakistan; d Amaris Consulting, Toronto, Ontario, Canada; e Aga Khan University Hospital, Karachi, Pakistan; f Department of Pediatrics, Children Hospital, Lahore, Pakistan; g Department of Pulmonology, Aga Khan University Hospital, Karachi, Pakistan

**Keywords:** COVID-19, Residual symptoms, Quality of life

## Abstract

**Background:**

There remains scarcity of literature regarding the patient's health status post-COVID-19 infection. This study analyzes the prevalence of residual symptoms and quality of life (QoL) after COVID-19.

**Methods:**

An anonymous online survey was administrated in Pakistan from November 2020 to April 2021 in COVID-19 survivors. The questionnaire used the 12-Item Short Form Health Survey (SF-12) to assess mental and physical QoL. Multivariate linear regression was used to explore factors associated with mental and physical QoL scores.

**Results:**

A total of 331 COVID-19 survivors participated in our survey. Around 42.0% of the cohort reported within 1–3 months of diagnosis of COVID-19. The common residual symptoms were body aches (39.9%), low mood (32.6%), and cough (30.2%). Better physical QoL was associated with being male (adjusted beta: 3.328) and having no residual symptoms (6.955). However, suffering from nausea/vomiting during initial COVID-19 infection (−4.026), being admitted to the ICU during COVID-19 infection (−9.164), and suffering from residual body aches (−5.209) and low mood (−2.959) was associated with poorer QoL. Better mental QoL was associated with being asymptomatic during initial COVID-19 infection (6.149) and post-COVID (6.685), while experiencing low mood post-COVID was associated with poorer mental QoL (−8.253 [-10.914, −5.592]).

**Conclusion:**

Despite presumed “recovery” from COVID-19, patients still face a wide range of residual symptoms months after initial infection, which contributes towards poorer QoL. Healthcare professionals must remain alert to the long-lasting effects of COVID-19 infection and aim to address them appropriately to improve patients’ QoL.

## Highlights

• COVID-19 survivors are currently facing the problem of “Long COVID Syndrome”.
• The syndrome is an illness that is cyclical, progressive, and multiphasic or multi-organ symptoms with the time duration varying from more than 1–2 months.
• Quality of Life of COVID-19 survivors is affected with this syndrome where the most common symptoms are fatigue and dyspnea.

## 1 Introduction

Coronavirus disease 2019 (COVID-19) was declared as a pandemic on March 11, 2020 by the World Health Organization (WHO) [1]. As of February 11, 2022, nearly 404 million people have contacted COVID-19 while the reported number of deaths stand at 5.7 million worldwide. After three waves of COVID-19 outbreaks, Pakistan currently has nearly 1.4 million confirmed cases and 29,000 deaths with its minister warning of a fourth wave if Standard Operating Procedures (SOPs) are not followed [1,2]. With a looming threat of yet another outbreak and a slow vaccination drive compared to the developed world, Pakistan is bound to experience continually rising case counts [3]. This growing number of “recovered” COVID-19 patients who eventually test negative for the disease are increasingly seen to face the problem of “Long COVID Syndrome”.

Though risk factors, clinical symptoms, management, and prognosis have been widely documented for patients with active COVID-19 infection, there remains uncertainty regarding the fate of patients who eventually test negative for COVID-19 after their infection. A lack of understanding in this regard warrants due investigation. Although the term ‘Long COVID’ is used to vaguely describe residual symptoms after COVID-19 infection, proper characterization of this condition is lacking [4]. Currently, it is best defined as an illness that is cyclical, progressive, and multiphasic or multi-organ symptoms with the time duration varying from more than 1–2 months of symptomatic presentation [4]. Other terms such as ongoing symptomatic COVID-19 (symptoms from 4 to 12 weeks) and post-COVID-19 syndrome (symptoms present for more than 12 weeks) are one of the many proposed terms to describe the condition [5].

The world witnessed previous outbreaks of coronavirus in the form of Middle East Respiratory Syndrome (MERS), where a lower QoL in survivors of critical illness was noted [6]. These findings are consistent with studies where lowered QoL have been reported in COVID-19 survivors as well. In addition to this, clinical symptoms of fatigue and dyspnea have been reported as the most common symptoms of Long COVID [7,8].

Given that only one study from Pakistan has reported on the issue, with a small sample size and non-diverse population, there is an urgent need for further research on the topic [9]. Thus, our study aims to investigate the prevalence, patterns, and risk factors of symptoms in survivors of COVID-19 infection, to report consistency, resolution, and emergence of symptoms a minimum of 1 month after diagnosed COVID-19 infection. Our study also aims to describe the QoL of patients who were at any point in time infected with COVID-19, and associate this with symptoms of Long COVID.

## 2 Methods

A large-scale, cross-sectional study was conducted in Pakistan to assess the residual symptoms and QoL in participants who had recovered from COVID-19 infection. The ethical review committee of Aga Khan University Hospital, Karachi (ERC Number: 2021-5584-17843) approved this study. The study is also registered on clinicaltrials.gov (NCT05148676). Using Google Forms, an anonymous online questionnaire was developed, using the official languages of Pakistan (Urdu and English), to be filled by adults (≥18 years old) who had been diagnosed with COVID-19 infection via polymerase chain reaction (PCR) testing ≥1 month ago from November 2020 till April 2021.

Previous validated questionnaires on residual symptoms after COVID-19 infection were adapted in consultation with subject experts, whereas the 12-Item Short Form Health Survey (SF-12) was used to assess QoL [6,8,10]. A pilot testing consisting of 30 participants was conducted, after which necessary changes were made to increase the clarity of the questions. Responses from the pilot testing were not included in the final analysis.

The sample size was calculated using OpenEpi (Version 3.01; Open-Source Epidemiologic Statistics for Public Health) which was estimated to be 384 participants (95% Confidence intervals (CI), bound on the error of 5%, and 50% acceptability). This was further increased to 400 participants to obtain maximum responses and compensate for any missing data. To counter social desirability bias, the investigators were kept anonymous with an online platform used for most of the responses.

Statistical analysis was performed using IBM SPSS Statistics for Windows, Version 23.0. Armonk, NY: IBM Corp. Continuous data was described using means and standard deviations and compared using independent sample t-tests. Categorical data was described using frequencies and percentages and compared using chi squared tests. The Mental Component Summary (MCS) and Physical Component Summary (PCS) scores were calculated as described in the questionnaire coding guide [11]. Univariate and multivariable linear regression was performed with the MCS and PCS scores as the dependent variables. Variables with p < 0.25 on the univariate model were included in the multivariate linear regression models. A p < 0.05 was considered significant for all analyses.

## 3 Results

A total of 331 individuals who responded to the online survey were included in the study. The majority were aged between 18 and 35 years (72.2%) and were residents of the province of Sindh (62.8%). Around half (54.1%) of the respondents were married, while 10.6% were separated or divorced. The highest level of education was reported as bachelors or lower by 45% of respondents. The common comorbidities were hypertension (9.4%), asthma (9.1%), and type 2 diabetes mellitus (7.3%). The clinicodemographic characteristics of the respondents are shown in Table 1.

**Table 1:** Respondents’ characteristics.

Variable	N (%)
**Age**	
18–35 Years	239 (72.2)
36–55 Years	68 (20.5)
> 55 Years	24 (7.3)
**Gender**	
Male	152 (45.9)
Female	179 (54.1)
**Marital Status**	
Married	179 (54.1)
Single (never married)	117 (35.3)
Separated/Divorced	35 (10.6)
**Province**	
Sindh	208 (62.8)
Punjab	103 (31.1)
Others	20 (6.1)
**Living Situation**	
Alone	36 (10.9)
Not Alone (Family/Roommate)	295 (89.1)
**Highest Level of Education**	
High School or Lower	75 (22.7)
Bachelors	149 (45.0)
Postgraduate Education	107 (32.3)
**Smoking Status**	
Current Smoker	21 (6.3)
Ex-Smoker	9 (2.7)
Never Smoked	301 (90.9)
**Comorbidities**	
Hypertension	31 (9.4)
Asthma	30 (9.1)
Type 2 Diabetes Mellitus	24 (7.3)
Depression	12 (3.6)
Immunodeficiency	9 (2.7)
Chronic Kidney Disease	2 (0.6)
Others	55 (16.6)

A total of 139 (42%) respondents reported that between 1 and 3 months had passed since they had been diagnosed with COVID-19, while 118 (35.6%) reported that more than 6 months had passed. The commonest symptoms during COVID-19 infection were fever (76.1%), cough (67.7%), body aches (65%), and an alteration in sense of smell (49.5%) or taste (44.1%). Only 5.1% of respondents reported being asymptomatic during COVID-19 infection. The commonest managements received were antipyretics (85.5%) and analgesics (58.3%). Steroids were received by 6.6% and remdesivir by 2.4% of respondents. Details regarding respondents’ COVID-19 history are provided in Table 2.

**Table 2:** COVID-19 history.

Variable	N (%)
**Time since COVID-19 Diagnosis**	
**1**–**3 Months**	139 (42.0)
**3**–**6 Months**	74 (22.4)
**≥ 6 Months**	118 (35.6)
**Symptoms during initial COVID-19 infection**	
Fever	252 (76.1)
Cough	224 (67.7)
Body Aches	215 (65.0)
Loss/Change in Sense of Smell	164 (49.5)
Loss/Change in Sense of Taste	146 (44.1)
Headache	143 (43.2)
Loss of Appetite	120 (36.3)
Nasal Stuffiness/Rhinorrhea	117 (35.3)
Diarrhea	87 (26.3)
Chest Discomfort/Pain	79 (23.9)
Hair Fall	74 (22.4)
Dyspnea	61 (18.4)
Nausea/Vomiting	59 (17.8)
Conjunctivitis	21 (6.3)
Other Symptoms	27 (8.2)
Asymptomatic	17 (5.1)
**Treatment/Management Received**	
Anti-Pyretic	283 (85.5)
Analgesics	193 (58.3)
Antibiotics	63 (19.0)
Steroids	22 (6.6)
Multivitamins	20 (6.0)
Ivermectin	17 (5.1)
Anti-Allergy	11 (3.3)
Remdesivir	8 (2.4)
Non-Pharmacologic (Steam/Oral Fluids)	13 (3.9)
No Treatment needed	28 (8.5)
**Residual Symptoms (Post-COVID-19)**	
Body aches	132 (39.9)
Low mood	108 (32.6)
Cough	100 (30.2)
Hair Fall	78 (23.6)
Headache	75 (22.7)
Loss of sense of smell	57 (17.2)
Loss of sense of taste	48 (14.5)
Loss of appetite	43 (13.0)
Chest Discomfort/Pain	42 (12.7)
Dyspnea	30 (9.1)
Fever	29 (8.8)
Nasal Stuffiness/Rhinorrhea	28 (8.5)
Diarrhea	27 (8.2)
Nausea/Vomiting	16 (4.8)
Conjunctivitis	2 (0.6)
Other Symptoms	33 (10.0)
Asymptomatic	54 (16.3)

The majority (83.7%) of respondents reported experiencing residual symptoms even >1 month after COVID-19 infection, with the most common symptoms being body aches (39.9%), low mood (32.6%), cough (30.2%), hair fall (23.6%) and headache (22.7%). The details of residual symptoms are given in Table 3. Cough as a residual symptom was significantly associated with time since COVID-19 diagnosis, with the highest prevalence (36.7%) amongst respondents 1–3 months post-COVID-19 infection and the lowest (21.2%) amongst those >6 months post-COVID-19 infection (p = 0.024). Headache, ageusia and anosmia were significantly associated with respondents’ age, with both being most common amongst respondents aged 18–35 years (25.9%, 14.2% and 17.2%, respectively) and 36–54 years (17.6%, 20.6% and 23.5%, respectively), and least common (4.2%, 0% and 0%, respectively) amongst respondents aged >55 years (p = 0.028, p = 0.047, and p = 0.032, respectively). Male respondents were also more likely to experience depression as a residual symptom, as compared to women (39.5% vs. 26.8%; p = 0.014). Respondents who were currently single were more likely to experience residual loss of appetite as compared to those who were married (17.8% vs. 8.9%; p = 0.017).

**Table 3:** Residual symptoms.

Residual Symptoms (>1-month post-COVID-19)	N (%)
**Body aches**	132 (39.9)
**Low mood**	108 (32.6)
**Cough**	100 (30.2)
**Hair fall**	78 (23.6)
**Headache**	75 (22.7)
**Loss/change in sense of smell**	57 (17.2)
**Loss/change in sense of taste**	48 (14.5)
**Loss of appetite**	43 (13.0)
**Chest Discomfort/Pain**	42 (12.7)
**Dyspnea**	30 (9.1)
**Fever**	29 (8.8)
**Nasal Stuffiness/Rhinorrhea**	28 (8.5)
**Diarrhea**	27 (8.2)
**Nausea/Vomiting**	16 (4.8)
**Conjunctivitis**	2 (0.6)
**Other Symptoms**	33 (10.0)
**Asymptomatic**	54 (16.3)

Respondents with pre-existing asthma were more likely to experience residual myalgia (63.3% vs. 37.5%; p = 0.006) and dyspnea (20% vs. 8%; p = 0.041), while current smokers were less likely to experience residual myalgia (19% vs. 41.3%; = 0.044). Respondents with hypertension were significantly more likely to experience a low mood post-COVID-19 infection (48.4% vs. 31%; p = 0.049), whereas respondents with pre-existing immunodeficiency were more likely to experience residual headache (55.6% vs. 21.7%; p = 0.031).

On multivariable linear regression with PCS as the dependent variable, being male (adjusted beta: 3.328 [95% Confidence Interval: 1.143, 5.513), >6 months since the time of COVID-19 infection diagnosis (3.192 [0.451, 5.932]), and being asymptomatic post-recovery from COVID-19 (6.955 [4.296, 9.614]) were independently associated with a better physical QoL. On the other hand, suffering from nausea or vomiting during initial COVID-19 infection (−4.026 [−6.886, −1.186]), being admitted to the ICU during COVID-19 infection (−9.164 [−16.168, −2.160]), and suffering from residual body aches (−5.209 [−7.170, −3.248]), low mood (−2.959 [−5.081, −0.838]), and chest discomfort (−4.842 [−7.738, −1.946]), was independently associated with poorer QoL (Table 4).

**Table 4:** Linear Regression identifying predictors of the PCS of SF-12.

Variable	Unadjusted Beta (SE)	95% CI	Adjusted Beta (SE)	95% CI
**Age**				
18–35 Years	Reference	–	Reference	–
36–55 Years	−2.002 (1.298)	*(-4.555, 0.551)*	NS	NS
> 55 Years	−1.071 (2.028)	(-5.062, 2.919)	–	–
**Gender**				
Female	Reference	–	Reference	–
Male	3.487 (1.038)	(1.444, 5.530) *	3.328 (1.111)	(1.143, 5.513) *
**Marital Status**				
**Married**	Reference	–	Reference	–
Single (never married)	0.344 (1.101)	(-1.822, 2.509)	–	–
Separated/Divorced	2.934 (1.794)	*(-0.418, 6.285)*	NS	NS
**Living Situation**				
Not Alone (Family/Roommate)	Reference	–	Reference	–
Alone	0.089 (1.690)	(-3.236, 3.415)	–	–
**Smoking Status**				
Current Smoker	2.513 (2.154)	*(-1.725, 6.752)*	NS	NS
Ex-Smoker	−3.636 (3.230)	(-9.989, 2.717)	–	–
Never Smoked	Reference	–	Reference	–
**Comorbidities**				
Hypertension	−5.006 (1.785)	(-8.518, -1.494) *	NS	NS
Asthma	−1.851 (1.830)	(-5.452, 1.749)	–	–
Type 2 Diabetes Mellitus	−5.371 (2.008)	(-9.320, -1.421) *	NS	NS
Depression	−3.412 (2.930)	(-9.716, 2.352)	–	–
Immunodeficiency	−1.800 (3.234)	(-8.162, 4.563)	–	–
Chronic Kidney Disease	−0.780 (6.791)	(-14.139, 12.578)	–	–
**Initial Symptoms**				
Fever	−2.290 (1.228)	*(-4.706, 0.126)*	NS	NS
Cough	−3.261 (1.116)	(-5.458, -1.065) *	NS	NS
Body Aches	−2.158 (1.097)	(-4.315, -0.001) *	NS	NS
Loss/Change in Sense of Smell	0.097 (1.053)	(-1.974, 2.168)	–	–
Loss/Change in Sense of Taste	−1.695 (1.056)	*(-3.772, 0.382)*	NS	NS
Headache	- 0.891 (1.060)	(-2.977, 1.195)	–	–
Loss of Appetite	−3.806 (1.074)	(-5.919, -1.692) *	NS	NS
Nasal Stuffiness/Rhinorrhea	−1.427 (1.098)	*(-3.587, 0.733)*	NS	NS
Diarrhea	−2.460 (1.183)	(-4.788, -0.132) *	NS	NS
Chest Discomfort/Pain	−4.221 (1.212)	(-6.606, -1.836) *	NS	NS
Hair Fall	-.3650 (1.247)	(-6.103, -1.196) *	NS	NS
Dyspnea	−3.170 (1.346)	(-5.818, -0.522) *	NS	NS
Nausea/Vomiting	−5.621 (1.340)	(-8.256, -2.985) *	−4.026 (1.443)	(-6.886, -1.186) *
Conjunctivitis	−3.372 (2.151)	*(-7.603, 0.859)*	NS	NS
Asymptomatic	4.376 (2.372)	*(-0.290, 9.042)*	NS	NS
Admitted to the Hospital	−3.536 (2.254)	*(-7.970, 0.898)*	NS	NS
Admitted to the ICU	−9.144 (3.623)	(-16.271, -2.017) *	−9.164 (3.560)	(-16.168, -2.160) *
**Time since COVID-19 Diagnosis**				
1–3 Months	Reference	–	Reference	–
3–6 Months	0.392 (1.263)	(-2.092, 2.877)	–	–
> 6 Months	3.863 (1.078)	(1.743, 5.984) *	3.192 (1.393)	(0.451, 5.932) *
**Residual Symptoms**				
Body Aches	−6.737 (1.009)	(-8.721, -4.753) *	−5.209 (0.997)	(-7.170, -3.248) *
Low Mood	−4.933 (1.089)	(-7.075, -2.791) *	−2.959 (1.078)	(-5.081, -0.838) *
Cough	−1.842 (1.142)	*(-4.088, 0.404)*	NS	NS
Hair Fall	−3.744 (1.223)	(-6.149, -1.338) *	NS	NS
Headache	−4.417 (1.233)	(-6.843, -1.990) *	NS	NS
Loss/Change in Sense of Smell	1.278 (1.392)	(-1.460, 4.017)	–	–
Loss/Change in Sense of Taste	0.054 (1.495)	(-2.886, 2.994)	–	–
Loss of Appetite	−2.553 (1.559)	*(-5.620, 0.514)*	NS	NS
Chest Discomfort/Pain	−6.854 (1.535)	(-9.874, -3.834) *	−4.842 (1.472)	(-7.738, -1.946) *
Dyspnea	−4.715 (1.815)	(-8.285, -1.146) *	NS	NS
Fever	−2.746 (1.855)	*(-6.395, 0.904)*	NS	NS
Nasal Stuffiness/Rhinorrhea	1.405 (1.890)	(-2.313, 5.122)	–	–
Diarrhea	−5.028 (1.903)	(-8.770, -1.285) *	NS	NS
Nausea/Vomiting	−3.457 (2.446)	*(-8.296, 1.356)*	NS	NS
Conjunctivitis	0.810 (6.791)	(-12.548, 14.169)	–	–
Asymptomatic	6.793 (1.374)	(4.090, 9.496) *	6.955 (1.352)	(4.296, 9.614) *

SE: Standard Error, CI: Confidence Interval, NS: Non-significant, ICU: Intensive Care Unit. *Statistically significant.

On multivariate linear regression with MCS as the dependent variable, being asymptomatic during initial COVID-19 (6.149 [0.630, 11.668]) infection and post-COVID (6.685 [3.470, 9.900]) were associated with better mental QoL, while experiencing low mood post-COVID was associated with poorer mental QoL (−8.253 [−10.914, −5.592]). In addition, being separated from one's partner or divorced was associated with poorer mental QoL (−5.658 [−10.276, −1.041]) (Table 5).

**Table 5:** Linear Regression identifying predictors of the MCS of SF-12.

Variable	Unadjusted Beta (SE)	95% CI	Adjusted Beta (SE)	95% CI
**Age**				
18–35 Years	Reference	–	Reference	–
36–55 Years	4.222 (1.548)	(1.177, 7.268) *	NS	NS
> 55 Years	5.285 (2.421)	(0.522, 10.048) *	NS	NS
**Gender**				
Female	Reference	**-**	Reference	**-**
Male	−0.216 (1.269)	(-2.713, 2.280)	NS	NS
**Marital Status**				
Married	Reference	**-**	Reference	–
Single (never married)	−2.662 (1.315)	(-5.249, -0.746) *	NS	NS
Separated/Divorced	−5.258 (2.036)	(-9.263, 1.252) *	−5.658 (2.347)	(-10.276, -1.041) *
**Living Situation**				
Not Alone (Family/Roommate)	Reference	**-**	Reference	–
Alone	4.448 (2.016)	(0.481, 8.415) *	NS	NS
**Smoking Status**				
Current Smoker	−4.005 (2.585)	*(-9.091, 1.080)*	NS	NS
Ex-Smoker	1.925 (3.887)	(-5.722, 9.572)	NS	NS
Never Smoked	Reference	–	Reference	–
**Comorbidities**				
Hypertension	0.484 (2.171)	(-3.786, 4.754)	–	–
Asthma	−2.750 (2.198)	*(-7.074, 1.573)*	NS	NS
Type 2 Diabetes Mellitus	0.631 (2.438)	(-.4166, 5.428)	–	–
Depression	−2.880 (3.535)	(-9.814, 4.054)	–	–
Immunodeficiency	2.944 (3.885)	(-4.699, 10.587)	–	–
Chronic Kidney Disease	−10.017 (8.142)	*(-26.034, 6.000)*	NS	NS
**Initial Symptoms**				
Fever	−3.447 (1.471)	(-6.342, -0.552) *	NS	NS
Cough	−1.715 (1.356)	*(-4.382, 0.951)*	NS	NS
Body Aches	−2.139 (1.320)	*(-4.736, 0.458)*	NS	NS
Loss/Change in Sense of Smell	−1.865 (1.261)	*(-4.345, 0.615)*	NS	NS
Loss/Change in Sense of Taste	−2.667 (1.265)	(-5.156, -0.178) *	NS	NS
Headache	−1.776 (1.272)	*(-4.278, 0.726)*	NS	NS
Loss of Appetite	−1.862 (1.312)	*(-4.442, 0.718)*	NS	NS
Nasal Stuffiness/Rhinorrhea	−3.008 (1.312)	(-5.590, -0.426) *	NS	NS
Diarrhea	−4.439 (1.410)	(-7.214, -1.665) *	NS	NS
Chest Discomfort/Pain	−4.030 (1.467)	(-6.916, -1.144) *	NS	NS
Hair Fall	−4.211 (1.500)	(-7.162, -1.260) *	NS	NS
Dyspnea	−1.429 (1.629)	(-4.634, 1.776)	–	–
Nausea/Vomiting	−3.713 (1.640)	(-6.939, -0.488) *	NS	NS
Conjunctivitis	−3.128 (2.589)	*(-8.221, 1.964)*	NS	NS
Asymptomatic	4.440 (2.855)	*(-1.176, 10.056)*	6.149 (2.805)	(0.630, 11.668) *
Admitted to the Hospital	1.764 (2.717)	(-3.581, 7.109)	–	–
Admitted to the ICU	0.391 (4.396)	(-8.256, 9.038)	–	**-**
**Time since COVID-19 Diagnosis**				
1–3 Months	Reference	–	–	–
3–6 Months	−0.471 (1.518)	(-3.456, 2.515)	–	–
> 6 Months	1.348 (1.318)	(-1.246, 3.941)	–	–
**Residual Symptoms**				
Body aches	−3.958 (1.273)	(-6.462, -1.454) *	NS	
Low mood	−9.153 (1.251)	(-11.613, -6.692) *	−8.253 (1.352)	(-10.914, -5.592) *
Cough	−0.891 (1.376)	(-3.599, 1.816)	–	–
Hair fall	−4.183 (1.472)	(-7.079, -1.287) *	NS	NS
Headache	−3.212 (1.500)	(-6.163, -0.261) *	NS	NS
Loss/Change in sense of smell	−0.682 (1.675)	(-3.976, 2.613)	–	–
Loss/Change in sense of taste	−1.979 (1.793)	(-5.505, 1.548)	–	–
Loss of appetite	−5.003 (1.861)	(-8.663, -1.342) *	NS	NS
Chest Discomfort/Pain	−3.421 (1.891)	*(-7.140, 0.299)*	NS	NS
Dyspnea	−3.817 (2.193)	*(-8.131, 0.496)*	NS	NS
Fever	−1.430 (2.235)	(-5.828, 2.967)	–	–
Nasal Stuffiness/Rhinorrhea	−0.289 (2.273)	(-4.759, 4.182)	–	–
Diarrhea	−4.235 (2.299)	*(-8.757, 0.287)*	NS	NS
Nausea/Vomiting	−4.000 (2.940)	*(-9.785, 1.784)*	NS	NS
Conjunctivitis	−0.744 (8.161)	(-16.797, 15.310)	–	–
Asymptomatic	7.282 (1.664)	(4.009, 10.555) *	6.685 (1.634)	(3.470, 9.900) *

SE: Standard Error, CI: Confidence Interval, NS: Non-significant, ICU: Intensive Care Unit. *Statistically significant.

## 4 Discussion

With only 3.5% of the Pakistani population being vaccinated, there has been a drastic increase in the number of active cases recently with the threat of a fourth wave [12]. As the COVID-19 cases continue to grow every day, the treatment for COVID-19 has evolved overtime allowing for a timely intervention improving the patient outcome. As COVID-19 survivors are released from the hospital, there is not enough literature which examines the long-term effects that COVID-19 has, physically and mentally, on these survivors. This cross-sectional study analyzes the post-COVID-19 symptoms and their impact on the QoL of recovered individuals.

A study by Zheng et al. reported the presence of palpitation, shortness of breath or dyspnea after physical activity in 56.3% of the discharged patients compared to our findings of 83.7% discharged patients also presenting with similar symptoms [13]. Since SARS-CoV-2 virus primarily targets the respiratory system, recovered individuals continue to experience shortness of breath up to 6 months due to abnormalities in the physiological functioning of lungs [14]. Upon examining the signs and symptoms of COVID-19 survivors in our study in categories such as OSC (4–12 weeks since initial infection) and PCS (>12 weeks), there were significant differences which could be associated with the functioning state of a patients’ immune system and age. Multivariate analysis of our data revealed dyspnea to be associated with a poor QoL. In addition to this, cough was reported with the highest prevalence amongst respondents 1–3 months post infection which dropped to 21.2% amongst those with more than 6 months since infection. No significant association was observed between cough and the QoL among our data. In a systematic review by Jennings et al., symptoms such as fever, chest pain and arthralgia decreased with time but during the PSC phase, the patients reported with an increase in dyspnea, palpitations, and myalgia [15]. Another observation that was made in our study was the presence of ageusia and anosmia most commonly in the age group 36–54 years with 20.6% and 23.5%, respectively. However, in patients with more than 55 years of age, ageusia and anosmia was not observed. These findings are in-aligned with another study from Korea which reported ageusia and anosmia to be present in the early stages of COVID-19 and more commonly in the age group like ours [16]. Some studies report that if anosmia and ageusia continue to persist more than a month then it might indicate permanent olfactory loss. This could be due to direct invasion by the virus on the olfactory bulbs through the trans neuronal route [17].

The most common post-COVID manifestation in our study was body aches reported by 39.9% of the participants. Similar follow-up studies were conducted in Italy and United Kingdom which also reported body aches to be one of the most prevalent post discharge symptoms [8,18]. There are other viral infections that usually lead to body aches are such as Epstein Barr virus and human immunodeficiency virus. Some studies have reported post-COVID patients to develop myalgia encephalomyelitis overtime characterized by diffuse muscle pain, depression, and sleep disturbances [19]. A meta-analysis was conducted on the long-term outcomes of recovered individuals where they reported body ache to be present in discharged patients at 18 months and 40 months in nearly one-third of the patients [20]. Univariate and multivariate analysis of our data revealed body aches to be associated with poor physical and mental QoL.

The second commonest residual symptom that was observed in our study was low mood reported by 32.6% participants followed by hair fall, headache, and loss of appetite. Upon multivariate analysis, all of these were associated with a poorer mental QoL. However, 16.3% of the participants were asymptomatic which showed a positive association with mental QoL. Further analysis of our data also revealed that male respondents were more likely to experience depression as compared to females, 39.5% versus 26.8%. A study conducted on the impact of mental health by COVID-19 consisting of 1000 respondents also observed males to be more depressed and stressed as compared to the females [21]. With the continual on-going COVID-19 pandemic, it has led to a ‘tsunami of psychiatric illness’ globally with long-lasting effects [21]. The social distancing and quarantine regulations have been reported to develop irritability, insomnia, and post-traumatic stress symptoms. The continual social isolation due to the COVID-19 pandemic along with the economic crisis can lead to the development of a phenomenon known as Hikikomori [21]. We reported 23.6% of the respondents to experience hair fall which been observed commonly in several survivors of COVID-19 across literature. This clinically relates to telogen effluvium which develops after an extended period of systematic stress and is often seen in the post-recovery period characterized by diffuse hair loss [22]. There can be several factors contributing to the physical and emotional stress some of which include sleep disturbance, financial issues, and fear of infection. Thus, in our data, low mood and hair fall was associated with a poor mental qualify of life.

Scientists have reported several similarities in the pathophysiological processes of SARS-CoV-1 and SARS-CoV-2 [23]. A similar follow-up study of 4 years was conducted for SARS infection where two common manifestations were observed in all recovered individuals, chronic fatigue and psychiatric conditions affecting the physical and mental well-being [23]. Both univariate and multivariate analysis in our data associated low mood with a poor physical and mental QoL. During the period of SARS infection or bubonic plague, a trend was observed with increased stigmatization among the communities [24]. The development of social disapproval due to the fear that the recovered individuals might still be contagious, affected many domains of everyday life for these survivors [24]. During this current COVID-19 pandemic, the exact same discrimination towards the survivors has put them at a higher risk of psychological problems. Therefore, dissemination of correct information through educational campaigns is imperative to create an environment based on biopsychosocial model.

Our study reports the largest sample size with the most diverse population from Pakistan to the best of our knowledge on the topic with nearly twice the number of participants compared to previous published literature. Haven translated the questionnaire to both the official languages of Pakistan, English and Urdu, this study enhanced the comprehensibility of the questionnaire ensuring accurate response to the questionnaire. Moreover, given some respondents might have experienced cognitive dysfunction in the effort to complete the survey, the questionnaire was designed as such as to not burden the participants hence the length and time taken to complete the questionnaire was kept succinct with no compromise on the quality of the questionnaire.

The retrospective nature of the study makes it vulnerable to recall bias that can impact the reliability of the results. Moreover, given the online nature of data collection, randomized sampling could not have been achieved alongside the possibility of under-representation of patients who might be in the ICU or in-patient setting. Additionally, given the questionnaire could have only been distributed online due to adherence to SOPs, participants who lacked digital literacy, internet or mobile phones, computers etc. may have been excluded. In future studies a more robust data collection process that ensures fair representation of severe cases alongside the population that lacks resources or education to respond to the questionnaire should be established to counter sampling bias.

## 5 Conclusion

To our knowledge, this study presents the longest follow-up symptom and QOL assessment after COVID-19 infection in Pakistani population. The findings of this study raise an alarming concern regarding the health consequences of discharged COVID-19 patients. The variability of post-COVID symptoms could be associated with the patients’ treatment, comorbidities, and age but further studies are needed to fully understand the underlying development of the symptoms. These long COVID symptoms show similarities to the symptoms experienced by SARS and MERS recovered individuals. A thorough analysis will allow for development of proper follow-up and management strategies in improving the physical and mental QoL outcomes in COVID-19 survivors.

## Available of data and materials

The datasets used and/or analyzed during the current study are available from the corresponding author on reasonable request.

## Author contributions

MAQ, RSM and RAD made substantial contributions to the conception and design of the work; AT, OI, QFS, WR, JAK and ASZ made substantial contributions or the acquisition, analysis, or interpretation of data; MAQ, RSM and RAD drafted the manuscript. All authors revised the manuscript critically and approved the final version and agree to be accountable for all aspects of the work in ensuring that questions related to the accuracy or integrity of any part of the work are appropriately investigated and resolved. All authors read and approved the final manuscript.

## Consent for publication

Not applicable.

## Registration of research studies

1. Name of the registry: Clinicaltrials.gov
2. Unique Identifying number or registration ID: NCT05148676
3. Hyperlink to your specific registration (must be publicly accessible and will be checked): https://clinicaltrials.gov/ct2/show/NCT05148676

## Funding

Authors received no financial support for conducting this research work.

## Ethical approval and consent to participate

The ethical review committee of Aga Khan University Hospital, Karachi (ERC Number: 2021-5584-17843) approved this study. Since data was collected through an online questionnaire, the consent of the participants was obtained before beginning the survey.

## Provenance and peer review

Not commissioned, externally peer reviewed.

## Guarantor

Dr. Rubaid Azhar Dhillon, M.B.B.S Department of Medicine, Medical College, Riphah International University, Email: rbazhar@hotmail.com Contact: +92310-000-1993.

## Declaration of competing interest

The authors declare that they have no competing interests.
